# Recommended reporting items for epidemic forecasting and prediction research: The EPIFORGE 2020 guidelines

**DOI:** 10.1371/journal.pmed.1003793

**Published:** 2021-10-19

**Authors:** Simon Pollett, Michael A. Johansson, Nicholas G. Reich, David Brett-Major, Sara Y. Del Valle, Srinivasan Venkatramanan, Rachel Lowe, Travis Porco, Irina Maljkovic Berry, Alina Deshpande, Moritz U. G. Kraemer, David L. Blazes, Wirichada Pan-ngum, Alessandro Vespigiani, Suzanne E. Mate, Sheetal P. Silal, Sasikiran Kandula, Rachel Sippy, Talia M. Quandelacy, Jeffrey J. Morgan, Jacob Ball, Lindsay C. Morton, Benjamin M. Althouse, Julie Pavlin, Wilbert van Panhuis, Steven Riley, Matthew Biggerstaff, Cecile Viboud, Oliver Brady, Caitlin Rivers

**Affiliations:** 1 Walter Reed Army Institute of Research, Silver Spring, Maryland, United States of America; 2 Division of Vector-Borne Diseases, Centers for Disease Control & Prevention, San Juan, Puerto Rico, United States of America; 3 University of Massachusetts–Amherst, School of Public Health and Health Sciences, Amherst, Massachusetts, United States of America; 4 University of Nebraska Medical Center, Omaha, Nebraska, United States of America; 5 Los Alamos National Laboratory, Los Alamos, New Mexico, United States of America; 6 Biocomplexity Institute and Initiative, University of Virginia, Charlottesville, Virginia, United States of America; 7 Centre for Mathematical Modelling of Infectious Diseases and Centre on Climate Change and Planetary Health, London School of Hygiene & Tropical Medicine, London, United Kingdom; 8 Barcelona Institute for Global Health, Barcelona, Spain; 9 University of California at San Francisco, San Francisco, California, United States of America; 10 Department of Zoology, University of Oxford, Oxford, United Kingdom; 11 Bill and Melinda Gates Foundation, Seattle, Washington, United States of America; 12 Mahidol-Oxford Tropical Medicine Research Unit and Department of Tropical Hygiene, Mahidol University, Thailand; 13 Network Science Institute, Northeastern University, Boston, Massachusetts, United States of America; 14 Modelling and Simulation Hub, Africa, Department of Statistical Sciences, University of Cape Town, Cape Town, South Africa; 15 Centre for Tropical Medicine and Global Health, Nuffield Department of Medicine, University of Oxford, Oxford, United Kingdom; 16 Department of Environmental Health Sciences, Mailman School of Public Health, Columbia University, New York City, New York, United States of America; 17 Institute for Global Health and Translational Science, State University of New York Upstate Medical University, Syracuse, New York, United States of America; 18 Catholic University of America, Washington, DC, United States of America; 19 U.S. Army Public Health Center, Edgewood, Maryland, United States of America; 20 Armed Forces Health Surveillance Division, Global Emerging Infections Surveillance, Silver Spring, Maryland, United States of America; 21 George Washington University, Milken Institute School of Public Health, Washington, DC, United States of America; 22 University of Washington, Seattle, Washington, United States of America; 23 Institute for Disease Modeling, Bellevue, Washington, United States of America; 24 New Mexico State University, Las Cruces, New Mexico, United States of America; 25 National Academies of Sciences, Engineering, and Medicine, Washington, DC, United States of America; 26 University of Pittsburgh Graduate School of Public Health, Pittsburgh, Pennsylvania, United States of America; 27 MRC Centre for Global Infectious Disease Analysis, School of Public Health, Imperial College, London, United Kingdom; 28 Influenza Division, Centers for Disease Control & Prevention, Atlanta, Georgia, United States of America; 29 Fogarty International Center, National Institutes for Health, Bethesda, Maryland, United States of America; 30 Centre for the Mathematical Modelling of Infectious Diseases, London School of Hygiene & Tropical Medicine, London, United Kingdom; 31 Johns Hopkins Center for Health Security, Johns Hopkins Bloomberg School of Public Health, Baltimore, Maryland, United States of America

## Abstract

**Background:**

The importance of infectious disease epidemic forecasting and prediction research is underscored by decades of communicable disease outbreaks, including COVID-19. Unlike other fields of medical research, such as clinical trials and systematic reviews, no reporting guidelines exist for reporting epidemic forecasting and prediction research despite their utility. We therefore developed the EPIFORGE checklist, a guideline for standardized reporting of epidemic forecasting research.

**Methods and findings:**

We developed this checklist using a best-practice process for development of reporting guidelines, involving a Delphi process and broad consultation with an international panel of infectious disease modelers and model end users. The objectives of these guidelines are to improve the consistency, reproducibility, comparability, and quality of epidemic forecasting reporting. The guidelines are not designed to advise scientists on how to perform epidemic forecasting and prediction research, but rather to serve as a standard for reporting critical methodological details of such studies.

**Conclusions:**

These guidelines have been submitted to the EQUATOR network, in addition to hosting by other dedicated webpages to facilitate feedback and journal endorsement.

## Abstract 2

Simon Pollett and co-workers describe EPIFORGE, a guideline for reporting research on epidemic forecasting.

## Introduction

The importance of infectious disease epidemic forecasting and prediction research is underscored across decades of communicable disease outbreaks. Epidemic forecasts are valuable for seasonal pathogens, for example, influenza and dengue [1–3], in addition to international health public emergencies and other epidemics such as the Zika, chikungunya, and Ebola virus epidemics [4–9]. Most recently, the Coronavirus Disease 2019 (COVID-19) pandemic has illustrated the importance of robust, transparent epidemic forecasting and prediction research for risk communication, decision-making, preparedness, and response [10,11]. Arguably, predictions form an essential part of the scientific method itself [12].

Other fields of medical research, such as clinical trials and systematic reviews, have widely used study reporting checklists, for example, the CONSORT and PRISMA guidelines [13]. Such checklists improve the interpretation, evaluation, and reproduction by other scientists and stakeholders, including public health decision-makers, journal editors, and journal reviewers. Indeed, many journals mandate that reporting checklists are completed prior to manuscript submission and publication, which has led to demonstrable improvements in study reporting [14,15]. Although principles for policy-driven communication of models for neglected tropical disease programs have been discussed [16], a recent systematic review noted no reporting guidelines exist specifically for epidemic forecasting and prediction research [17]. The need for epidemic forecasting reporting guidelines is underscored by a review of Zika forecasting and prediction research, which noted methodological reproducibility, accessibility, and incorporation of uncertainty in these published predictions varied [8].

To address this gap, we developed the EPIFORGE checklist, the first known set of epidemic forecasting reporting guidelines. This checklist was developed through a well-established process for developing guidelines for research reporting, involving a Delphi process and broad consultation with an international panel of infectious disease modelers and model end users [18,19]. The objectives of these guidelines are to improve the consistency, reproducibility, comparability, and quality of epidemic forecasting reporting. Here, we describe our guidelines development process and the resulting checklist. The EPIFORGE checklist is not designed to advise scientists how to perform epidemic forecasting and prediction research, but rather serve as a set of standards to ensure critical aspects of these studies are reported in a standardized way.

## Methods

We followed health research reporting guideline development best practice as outlined in the EQUATOR toolkit and by Moher and colleagues [18,19] and summarized in full in the Supporting information (S1 Text). Briefly, The EPIFORGE guideline concept was registered at the EQUATOR network, and a steering committee (*n =* 6) formed to develop a guideline development protocol. Members from this steering committee had already identified a case study that prompted the need for EPIFORGE and conducted a systematic review to ensure no epidemic forecasting reporting guideline existed [17]. The EPIFORGE steering committee formulated an initial draft checklist of 20 reporting items during 2 teleconferences. This draft checklist was the input for an iterative Delphi consensus process. A total of 69 Delphi panelists were invited, and 46 participated in this process (S1 Table). During 3 initial rounds of Delphi consultations via email (September, October, and December 2019), panelists graded each checklist item on a scale of 1 through 10 (a score of 1 was defined as “not important,” and a score of 10 was defined as “very important”), with an emphasis on voting based on the concept of the item (rather than the wording). Checklist items with a mean score ≥8 were retained for the final reporting checklist, items with a mean score <5 were dropped, and items with a mean score 5 to 7 were kept for further discussion at a final face-to-face consensus meeting (January 2020). Additional items were added by Delphi participants during the first 2 email Delphi rounds.

## Results

Table 1 presents the final consensus checklist items, including reporting elements on study goals, data sources, model characteristics and assumptions, model evaluation, and study generalizability. Below, we elaborate and explain each item:

### A. Overall study description and goals

#### Item 1: Describe the study as forecast or prediction research in at least the title or abstract

These guidelines primarily refer to forecasting research, but the principles are applicable to prediction research more broadly and can be used for these other study types. Forecasting research has been defined as research that “*typically offers quantitative statements about an event*, *outcome*, *or trend that has not yet been observed*, *conditional on data that has been observed*,” whereas prediction research is broader and has been defined as a field, which “*may refer to models that examine the mechanistic drivers of epidemiological characteristics …*. *as well as studies that estimate epidemiological characteristics with inherent forecasting value*, *such as R*_*0*_” [17]. As the 2 terms may be conflated by studies, we recommend that the study is described as a forecast or prediction research in at least the title or abstract (for example, [20–22]). While limiting to the terms “forecast,” “forecasting,” or “prediction” may be too restrictive, we believe that limiting the number of terms is important to enable findability (accurate returns on searches) in the literature and may assist in standardizing nomenclature across the field.

Epidemic forecasts may be conflated with projections, simulations, or scenario analyses [4,10,20], which may or may not be fundamentally different in nature. Here, we refer to forecasts and predictions as predictions of what *will* happen. Other valuable research focuses on projections, simulations, or scenario analyses, which can be framed as “what if” scenarios, i.e., what would happen *conditionally* under certain conditions or assumptions (including, for example, assumptions of no interventions or no control of an epidemic, or assumptions of long-term seasonality and varied population immunity) [23,24]. Forecasts often refer to more shorter-term predictions [23]. Many of the reporting principles in this guideline may also be useful for projections, simulations, or scenario analyses.

#### Item 2: Define the purpose of study and forecasting targets

Clearly identifying the research objectives is a fundamental element of any scientific study and is a feature of many other research reporting guidelines [19]. We recommend that forecasting targets (i.e., each specific observable outcome being forecasted such as a 2-week-ahead incidence, peak week, observation of at least 1 case) should be defined in the introduction section, and, ideally, also in the abstract (for example, [21,22,25]).

#### Item 3: Fully document the methods

Methods documentation is essential to any scientific study and follows general best practice for the reporting of other research study types [19]. We recommend that forecasting methods should include a full description of the model that enables reproducibility, the method of fitting parameters to data (for example, maximum likelihood with function if nonstandard, Bayesian methods), and—where relevant—underlying epidemic model assumptions (see also **Item 8**). For example, [20].

#### Item 4: Identify whether the forecast was performed prospectively, in real time, and/or retrospectively

We recommend that it is identified whether the forecast was performed prospectively, in real time, and/or retrospectively (for example, [22]). This item is necessary for interpreting results of forecasting accuracy and may aid in determining whether authors were blinded to a hold-out set (out-of-sample set) of data used for any model validations. See also **Item 16** for recommendations on time-stamping the results of forecasts.

### B. Data description

#### Item 5: Explicitly describe the origin of input source data, with references

We recommend that the origin of the input source data is provided (for example, [21,26]). This item is essential for study reproducibility and is a minimum requirement for any manuscript, even if full study data cannot be publicly shared (see **Item 6**). For all data types—including laboratory assay, case counts, demographic data, and nontraditional data streams (for example, internet event-based data signals)—the authors should include sufficient references to be able to identify the input data [27] and ideally a persistent and unique identifier that resolves to the (meta)data (for example, [6]).

#### Item 6: Provide source data with publication, or document reasons as to why this was not possible

We recommend that source data are made available. Provision of source data improves forecast reproducibility. Sharing of source data used in forecasts (for example, [1]) facilitates other complementary studies, including those which may independently validate forecasts and methods. Limitations on data sharing during epidemics is a known challenge [28]. We are aware of efforts to establish codes of conduct for data sharing during public health emergencies [29] but recognize the wide range of logistical and other barriers to data sharing during outbreaks [28,30]. Therefore, we suggest at a minimum reporting of the reasons for not providing source data with forecast publication. Several major biomedical journals now routinely require authors to provide deidentified data [31]. When data are provided, we recommend inclusion of a data dictionary and/or structured metadata in a standardized format.

#### Item 7: Describe input data processing procedures in detail

We recommend that the input data processing procedures are described in detail (for example, [21,22]). This is an important feature for study reproducibility. Preprocessing procedures may include recoding and imputation of missing observations, identification and management of extreme outliers and influential data points, and functional transformations such as data normalization. Provision of data preprocessing code may also be useful.

### C. Model characteristics

#### Item 8: State and describe the model type, and document model assumptions, including references

We recommend that the model type is stated and described, and the model assumptions documented (for example, [21,22]). This is critical for study reproducibility, and it allows interpretation of model output in the context of any assumptions presented. Describing model parameter values and assumptions, with references, further allows other researchers to use cited parameter values in their own work (after careful consideration), and this may expedite forecasting efforts in a public health emergency. For an ongoing epidemic, if the model makes specific assumptions about current and future interventions and their impact, they need to be stated with appropriate justification. Model types may include mechanistic or statistical representation of disease transmission; models may also be classified as stochastic or deterministic models. These are just some of several classifications of model types [32]. We do not propose a categorization scheme for model types in these guidelines due to the wide range of model type nomenclature that is often heterogeneously used by modelers. Developing such a schema could be the subject of future research.

#### Item 9: Make the model code available, or document the reasons why this is not possible

We recommend that model code is made available. Providing model code improves research reproducibility, especially if accompanied by documentation, and may facilitate the rapid conduct of other studies addressing the same or similar study question(s), especially during a public health emergency. Some forecasting studies already have provided model code during public health emergencies of international concern [6,33]. Infectious disease modelers have also made the point that publication of model code may permit direct comparisons of model performance in real time by external groups [7]. We emphasize that providing model code is strongly encouraged. There are valid reasons for why researchers may not be able to provide model code, including possible intellectual property concerns (this potential consideration has been illustrated in the artificial intelligence modeling field more broadly, for example, [34]), or specific concerns about potential misuse (i.e., using the forecasting model as a “black box” without understanding its principles and limitations). In cases where code is not made available, we propose that authors provide a brief justification for why this is the case. This may assist in future studies that seek to identify and mitigate barriers to sharing forecast model code during public health emergencies. A clear statement of model code availability will also allow journals to screen submissions for this feature.

### D. Model evaluation

#### Item 10: Describe the model validation, and justify the approach

We recommend describing the model validation method and justifying the approach. Forecast model validation is critical to ensure accuracy of results and usefulness of models, and it also encourages trust in the results and methods by other researchers, journal reviewers, journal editors, and end users. Forecasting research should indicate if cross-validation or out-of-sample validation was performed, the data used for validation, how many models were considered at each stage of validation, the time span of validation (with justification), and whether the researchers were blinded to the external validation dataset (for example, through a prospective design like a forecast challenge or other real-time forecasting exercise) [2,5,35–37].

#### Item 11: Describe the forecast accuracy evaluation method used, with justification

We recommend that the forecast accuracy evaluation method is described and justified. Forecast and prediction research studies may include point predictions (for example, mean number of expected cases) or a full probability distribution of the outcome of interest. It is important that the metric of validation accuracy is both clearly defined and justified, thereby allowing forecast performance to be robustly evaluated and compared between studies when using the same data. Examples include [22].

#### Item 12: Where possible, compare results to a benchmark or other comparator model, with justification of comparator choice

We recommend that the forecast results are compared to a benchmark or other comparator model, and the comparator model choice justified. Benchmark models may include relatively simple models such as autoregression or seasonal averages [38]. These comparisons are important to mitigate the risk of model misspecification and may also provide a “common sense” interpretation of forecast value compared to intuitive benchmarks such as an autoregression model with a 1-week lag time [39–41]. If there are other published models for the specific forecasting target or type of target that demonstrate significant improvement compared to simpler models, those forecasts should be used as the comparator to the extent possible [20]. Comparison may include formal statistical comparisons with established methods (for example, Diebold-Mariano tests or permutation tests) [42,43]. For emerging pathogens with novel disease traits and novel forecasting targets, such benchmark models may not be readily available.

#### Item 13: Describe the forecast horizon, with justification of its length

We recommend that the forecast horizon is described, and its length justified (for example, [21]). Presenting forecast accuracy and precision with increasing lead times allows for an evaluation of a forecast’s usefulness over operationally relevant timescales. We suggest justification of the forecast horizon to avoid inadvertent misrepresentation of model accuracy and precision and to communicate the inherent limits of forecasts that may break down over longer forecast horizons [38,41].

#### Item 14: Present and explain uncertainty of forecasting results

We recommend that uncertainty in the forecasting results is presented and explained. Uncertainty is a fundamental consideration in developing and interpreting epidemic forecasting and prediction research. Uncertainty can arise from parameters, assumptions, model choice, lack of knowledge about the epidemiology of the disease, or variability in the data itself. Qualitative and/or quantitative estimates of uncertainty can be incorporated into forecasting research through using probabilistic forecast methods, uncertainty intervals around point estimates (for example, prediction or credibility intervals), sensitivity or scenario analyses, or description of the uncertainty in the model parameters. We recommend that the estimates of uncertainty are clearly described in at least the results, and, ideally, also referred to in the discussion and the abstract. Examples include [20,22].

### E. Translation of results for public health practice, interpretability, and generalizability

#### Item 15: Briefly summarize the results in nontechnical terms, including a nontechnical interpretation of forecast uncertainty

We recommend that the results are summarized in nontechnical terms. Adequately reporting and explaining model forecasts is critical for a wide range of readers, including public health decision-makers and the media. Forecasts can be misinterpreted, especially when uncertainty is not explicitly and clearly communicated with a broad audience in mind. We propose that a lack of appropriate communication about these inherent caveats in forecasting science may lead to skepticism of forecasting by important end users (such as decision-makers), the media, and the general public. We recommend a brief nontechnical summary of forecasting research results, as already required by several major biomedical journals for a range of research fields [44], and including a nontechnical interpretation of forecast uncertainty. Examples include [45,46].

#### Item 16: If results are published as a data object, encourage a time-stamped version number

In general, we recommend that results, i.e., the raw forecast data themselves and summaries of these data, are made available as a public data object. This reporting recommendation serves multiple purposes. First, it allows searching and aggregating of forecast results by a standardized object nomenclature. Second, it ensures that forecasts are truly prospective, when claimed to be so. Third, it permits clear communication of when forecasts are updated (for instance, as parameter estimates are refined, or as new data becomes available). We recommend assigning a unique and persistent identifier to the time-stamped and versioned data object, such as a digital object identifier (DOI). This practice could extend to web-based forecasting tools linked to the publication also.

#### Item 17: Describe the weaknesses of the forecast, including weaknesses specific to data quality and methods

We recommend that the weaknesses in the forecast be described. Limitations can include data quality (for example, heterogeneity in sampling over time and across populations, diagnostic limitations, or case selection bias), parameter uncertainty, model misspecification, or limitations in generalizability. No model is a complete representation of reality, and much can be gleaned about a forecasting model’s utility from knowing its limitations or simplifying assumptions. It is important to note that identifying methodological weaknesses in forecasts does not necessarily mean that they lack credibility. Rather, highlighting such weaknesses may inform data needs, lead to improvements of forecasts, and assist in interpretation of forecast results during public health decision-making. For example, [22,46].

#### Item 18: If the forecast research is applicable to a specific epidemic, comment on its potential implications and impact for public health action and decision-making

When forecasting research is intended to be applicable to a specific outbreak or epidemic, we propose that the potential implications of the forecast for that specific epidemic need to be described, including whether it has a possible impact on public health action or decision-making. Framing the discussion of results in this context is essential for model end users and may assist in ensuring that model developers are addressing the right research questions from the outset. For example, [22].

#### Item 19: If the forecast research is applicable to a specific epidemic, comment on how generalizable it may be across populations

When forecasting research is intended to be applicable to a specific outbreak or epidemic, researchers should describe the generalizability of results between countries, regions, populations, and perhaps even pathogens, together with the rationale for why (for example, [22]). A forecast’s accuracy or applicability in one setting may not translate to others due to inherent differences in healthcare capacity, population demography, disease ecology, socioeconomic factors, and data availability and reliability.

**Table 1:** EPIFORGE 2020 checklist.

Section of manuscript	#	Checklist item	Reported on page^a^
Title/Abstract	1	Describe the study as forecast or prediction research in at least the title or abstract	
Introduction	2	Define the purpose of study and forecasting targets	
Methods	3	Fully document the methods	
Methods	4	Identify whether the forecast was performed prospectively, in real time, and/or retrospectively	
Methods	5	Explicitly describe the origin of input source data, with references	
Methods	6	Provide source data with publication, or document reasons as to why this was not possible	
Methods	7	Describe input data processing procedures in detail	
Methods	8	State and describe the model type, and document model assumptions, including references	
Methods	9	Make the model code available, or document the reasons why this was not possible	
Methods	10	Describe the model validation, and justify the approach	
Methods	11	Describe the forecast accuracy evaluation method used, with justification	
Methods	12	Where possible, compare model results to a benchmark or other comparator model, with justification of comparator choice	
Methods	13	Describe the forecast horizon, with justification of its length	
Results	14	Present and explain uncertainty of forecasting results	
Results^b^	15	Briefly summarize the results in nontechnical terms, including a nontechnical interpretation of forecast uncertainty	
Results	16	If results are published as a data object, encourage a time-stamped version number	
Discussion	17	Describe the weaknesses of the forecast, including weaknesses specific to data quality and methods	
Discussion	18	If the research is applicable to a specific epidemic, comment on its potential implications and impact for public health action and decision-making	
Discussion	19	If the research is applicable to a specific epidemic, comment on how generalizable it may be across populations	

^a^This column refers to where key reporting considerations are included in a manuscript.

^b^A break-out box may be a preferred location.

## Conclusions

We present the first guidelines for standard reporting of epidemic forecasting research, comprising 19 preferred items in a checklist. We stress that the objectives of these guidelines are intended to improve the epidemic forecasting reporting consistency and reproducibility, as well as comparability and quality. They serve as a set of standards to ensure that critical aspects of these studies are adequately reported and are not intended to advise scientists on how to perform epidemic forecast and prediction research. We note that our Delphi process also led to several checklist items, which pertain to the translation of forecasting results for public health practice.

The primary target audience of these guidelines is scientists using models to forecast infectious disease epidemics as a means to ensure that critical reporting items are included in published manuscripts. While this checklist may also serve as a means of ensuring standardization of infectious disease modeling quality among this group, it is distinct from other structured consensus documents, which have focused on modeling principles or made recommendations for reporting of other types of modeling studies [47–50]. The secondary target audience of these guidelines include model users (for example, those in operational public health and policy), journal peer reviewers, journal editors, and epidemiology training programs. We encourage formal endorsement by modeling groups and broad adoption by biomedical journals who already require completion of reporting checklists for manuscript submissions, including clinical trials and systematic reviews [51]. While our guidelines were developed with peer-reviewed published research papers in mind, these could be applied to epidemic forecasting research reported elsewhere.

Research reporting guidelines do need to be subfield specific to be pragmatic and useful (for this reason the EQUATOR website references over 440 guidelines), but it is worth comparing our final guidelines to others medical reporting guidelines, which have been widely implemented, such as CONSORT and PRISMA [14]. Like EPIFORGE, these guidelines identify the study type, define the study objectives, comment on study limitations, aid in interpreting the validity of the results, and discuss generalizability of the findings.

Our approach to development of this checklist has some limitations. While the major strength of the EPIFORGE guidelines is the use of a structured Delphi process across a range of stakeholders, this resulted in a number of valuable reporting considerations suggested by the Delphi panel, which were not included after the consensus process. We noted several items suggested by the Delphi panel that were not ultimately voted in. These covered a range of topics and may not be applicable to all forecasting and prediction research. We include these items as a supporting appendix for general consideration in the field of reporting forecasting and prediction research, and these may be reconsidered in future versions of the EPIFORGE reporting guidelines (S2 Table). Future versions should also seek to identify other items which through a new Delphi process.

While the development process involved broad consultation, we encourage broad and frank feedback and critique. Feedback will be valuable in updating future iterations of these guidelines, which are intended to be dynamic and responsive to the ongoing needs of epidemic forecasters and end users, including those involved in COVID-19 research and response. These guidelines have been submitted to the EQUATOR Network webpage, in addition to dedicated webpages to facilitate feedback and journal endorsement ([20–22]; https://midasnetwork.us/), following examples from other guidelines [14].

## Supporting information

S1 Text Full description of methods. (DOCX)

S1 Table Delphi panel members. (DOCX)

S2 Table Other reporting considerations not included into the final reporting checklist. (DOCX)
