# Correction: Recommended reporting items for epidemic forecasting and prediction research: The EPIFORGE 2020 guidelines

**DOI:** 10.1371/journal.pmed.1004316

**Published:** 2023-11-17

**Authors:** Simon Pollett, Michael A. Johansson, Nicholas G. Reich, David Brett-Major, Sara Y. Del Valle, Srinivasan Venkatramanan, Rachel Lowe, Travis Porco, Irina Maljkovic Berry, Alina Deshpande, Moritz U. G. Kraemer, David L. Blazes, Wirichada Pan-ngum, Alessandro Vespigiani, Suzanne E. Mate, Sheetal P. Silal, Sasikiran Kandula, Rachel Sippy, Talia M. Quandelacy, Jeffrey J. Morgan, Jacob Ball, Lindsay C. Morton, Benjamin M Althouse, Julie Pavlin, Wilbert van Panhuis, Steven Riley, Matthew Biggerstaff, Cecile Viboud, Oliver Brady, Caitlin Rivers

There is an error in reference 30. The correct reference is: Rivers C, Chretien JP, Riley S, Pavlin JA, Woodward A, Brett-Major D, et al. Using "outbreak science" to strengthen the use of models during epidemics. Nat Commun. 2019;10(1):3102. doi: 10.1038/s41467-019-11067-2. PMID: 31308372.
