# Supplementary material for: Recommended reporting items for epidemic forecasting and prediction research: The EPIFORGE 2020 guidelines
Source: PLoS Med. 2021 Oct 19;18(10):e1003793. doi: 10.1371/journal.pmed.1003793 (PMC8525759; doi:10.1371/journal.pmed.1003793)
Supplement: S1 Text — (DOCX) [file pmed.1003793.s001.docx]

**Supplementary Material: Full Methods**

The EPIFORGE guideline concept was registered at the EQUATOR network, and a steering committee (n = 6) formed to develop a guideline development protocol [1]. Members from this steering committee had already identified a case study that prompted the need for EPIFORGE [2], and conducted a systematic review to ensure no epidemic forecasting reporting guideline existed [3]. The EPIFORGE steering committee formulated an initial draft checklist of 20 reporting items during two teleconferences. This draft checklist was the input for an iterative Delphi consensus process as used in other research reporting guidelines [4]. A total of 69 Delphi panelists were invited, and 46 participated in this process. The Delphi panel comprised infectious disease modelers, public health experts who routinely use epidemic forecasts in public health practice, epidemiologists, and biomedical journal editors across several countries (Appendix S1). The candidate panelists were selected by the steering committee to incorporate the perspectives of those who both develop and use models across a range of sectors, including academia, government, and non-government organizations around the globe. Some panelists who were invited further suggested other potential panelists.

During three initial rounds of Delphi consultations via email, panelists graded each checklist item on a scale of 1 through 10 (a score of 1 was defined as “not important”, and a score of 10 was defined as “very important”), with an emphasis on voting based on the concept of the item (rather than the wording). Checklist items with a mean score ≥ 8 were retained for the final reporting checklist, items with a mean score <5 were dropped, and items with a mean score 5 – 7 were kept for further discussion at a final face-to-face consensus meeting. Additional items were added by Delphi participants during the first two email Delphi rounds. In addition, for each round, panelists were invited to provide comments about the wording of the item, provide a rationale for their vote, and provide citations of evidence to support any new items.

All 46 Delphi panelists were invited to a face-to-face consensus meeting in Baltimore, Maryland (January, 2020). 20 panelists attended either in person or remotely by live video conference. The purpose of the meeting was to discuss intermediately scored items (mean score 5 – 7) and vote on them. Those items with a simple majority were included in the final reporting checklist. During this meeting, suggestions about the final wording, and consolidation of similar items, were discussed and documented. The steering committee then drafted a final version of the checklist. This was sent back to all in-person attendees for final comments, and participants had opportunity to ‘pilot test’ the checklist during their own epidemic forecasting activities, including COVID19 forecasting (this invitation for pilot testing yielded no changes to these guidelines). This checklist, along with this elaboration and explanation paper, was then provided to the full Delphi panel for final review and endorsement. During these final reviews, we also requested examples of already published epidemic forecasting papers to illustrate the reporting of specific items.

[1] The EQUATOR Network | Enhancing the QUAlity and Transparency Of Health Research. [cited 13 Aug 2021]. Available: <https://www.equator-network.org/>

[2] Kobres P-Y, Chretien J-P, Johansson MA, Morgan JJ, Whung P-Y, Mukundan H, et al. A systematic review and evaluation of Zika virus forecasting and prediction research during a public health emergency of international concern. Pimenta PFP, editor. PLoS Negl Trop Dis. 2019;13: e0007451. doi:10.1371/journal.pntd.0007451

[3] Pollett S, Johansson M, Biggerstaff M, Morton LC, Bazaco SL, Brett Major DM, et al. Identification and evaluation of epidemic prediction and forecasting reporting guidelines: A systematic review and a call for action. Epidemics. 2020;33: 100400. doi:10.1016/j.epidem.2020.100400

[4] Moher D, Schulz KF, Simera I, Altman DG. Guidance for Developers of Health Research Reporting Guidelines. PLOS Med. 2010;7: e1000217. doi:10.1371/journal.pmed.1000217
