# Supplementary material for: Recommended reporting items for epidemic forecasting and prediction research: The EPIFORGE 2020 guidelines
Source: PLoS Med. 2021 Oct 19;18(10):e1003793. doi: 10.1371/journal.pmed.1003793 (PMC8525759; doi:10.1371/journal.pmed.1003793)
Supplement: S1 Table — (DOCX) [file pmed.1003793.s002.docx]

| **Table S1. Delphi panelists who participated in the EPIFORGE development (listed alphabetically by first name)** | |
| --- | --- |
| Participant | Organization |
| Adam Kucharski | London School of Hygiene & Tropical Medicine, UK |
| Alessandro Vespignani | Northeastern University, USA |
| Alina Deshpande | Los Alamos National Laboratory, USA |
| Anne Cori | Imperial College London, UK |
| Benjamin Althouse | University of Washington, USA |
| Benjamin Ervin | Massachusetts Institute of Technology, USA |
| Bryan Lewis | University of Virginia, USA |
| Caitlin Rivers | Johns Hopkins Center for Health Security, USA |
| Cécile Viboud | Fogarty International Center at National Institutes of Health, USA |
| David Blazes | Bill and Melinda Gates Foundation, USA |
| David Brett Major | University of Nebraska Medical Center, USA |
| Dylan George | In-Q-Tel, USA |
| Helen Johnson | European Centre for Disease Prevention and Control, Sweden |
| Henrik Salje | University of Cambridge, UK |
| Irina Maljkovic Berry | CNTS for Walter Reed Army Institute of Research, USA |
| Jacob Ball | Army Public Health Center, USA |
| Jason Asher | HHS Office of the Assistant Secretary for Preparedness and Response, USA |
| Jay Varma | Africa Centers for Disease Control and Prevention, Ethiopia |
| Jean-Paul Chrétien | US Department of Defense, USA |
| Jeffrey J Morgan | Catholic University of America, DC, USA |
| Julie Pavlin | National Academies of Sciences, Engineering, and Medicine, USA |
| Katelijn Vandemaele | World Health Organization, Switzerland |
| Lindsay Morton | Armed Forces Health Surveillance Branch, USA |
| Macarena Garcia | Centers for Disease Control and Prevention, USA |
| Matthew Biggerstaff | Center for Disease Control and Prevention, USA |
| Michael Johansson | Centers for Disease Control and Prevention, USA |
| Moritz Kraemer | University of Oxford, UK |
| Nicholas Reich | University of Massachusetts Amherst, School of Public Health & Health Sciences, USA |
| Oliver Brady | London School of Hygiene & Tropical Medicine, UK |
| Rachel Lowe | London School of Hygiene & Tropical Medicine, UK |
| Rachel Sippy | State University of New York – Upstate, USA |
| Raina MacIntyre | University New South Wales, Australia |
| Travis Porco | University of California San Francisco, USA |
| Roni Rosenfeld | Carnegie Mellon University, USA |
| Cathy Roth | Department for International Development, UK |
| Sara Del Valle | Los Alamos National Laboratory, USA |
| Sasikiran Kandula | Columbia University, Mailman School of Public Health, USA |
| Mark Scheckelhoff | Armed Forces Health Surveillance Branch, USA |
| Sheetal Silal | University of Cape Town, South Africa |
| Simon Pollett | CNTS for Walter Reed Army Institute of Research, USA |
| Srinivasan Venkatramanan | University of Virginia, USA |
| Steven Riley | Imperial College of London, UK |
| Suzanne Mate | Walter Reed Army Institute of Research, USA |
| Talia Quandelacy | Centers for Disease Control and Prevention, USA |
| Wilbert Van Panhuis | University of Pittsburgh Graduate School of Public Health, USA |
| Wirichada Pan-ngum | Mahidol University, Thailand |
