# Supplementary material for: Recommended reporting items for epidemic forecasting and prediction research: The EPIFORGE 2020 guidelines
Source: PLoS Med. 2021 Oct 19;18(10):e1003793. doi: 10.1371/journal.pmed.1003793 (PMC8525759; doi:10.1371/journal.pmed.1003793)
Supplement: S2 Table — (DOCX) [file pmed.1003793.s003.docx]

| **Table S2. EPIFORGE checklist items which were not included in the final reporting guidelines^a^** |
| --- |
| Metadata file with minimal metadata elements for forecast data object published |
| Method of generation/curation of special data sources such as internet big data, genomic data or serological data explicitly described with reference as required |
| Clearly worded conditions necessary for forecast |
| Method of blinding of forecast developers to out-of-sample external validation dataset described |
| Quantify how forecast accuracy varies with increasing lead times |
| Disaggregate forecast accuracy by sensitivity and specificity (as consequences of false positive and false negative are rarely equal) |
| Clear definition of evaluation metric that matches with point or probabilistic forecasts (i.e. if probabilistic forecasts are made, but only MSE is used to evaluate, this is a mismatch) |
| Clearly state the number of iterations the model was used to demonstrate prediction value over time. For seasonal epidemics, require the forecast run throughout the epidemic and non-epidemic cycle, with results of each iteration made visible as supplemental data. The results section should address forecast performance with real-time events (in-borne validation strategy) |
| Method of calibration to data (e.g. MLE, MCMC) and fitting metric (e.g. least-squares, parametric observation process) |
| Lay-term explanation of model equation |
| Rationale for model type (e.g. deterministic vs stochastic) justified |
| Credible intervals provided |
| Clear statement about whether forecasts made are probabilistic or point. (ideally in abstract) |
| Presentation of forecast skill using standard metrics (e.g. proper score or error metrics) relative to standard or alternative models |
| Data needs (additional data that would have improved the forecasts, but was unavailable for some reason)” |
| Ability to update forecasts for ongoing outbreaks |
| List of previous models on topic (to assure adequate literature review) |
| Description of whether forecast published/to be published as an open access pre-print with rationale for decision |
| Stakeholder engagement process and consultation described (e.g. how were forecasts presented to end-users and reiterated given feedback) |
| Were the results communicated to health agencies/NGOs/other partners, and when |
| Affiliation of authors and current funding mechanism described |
| ^a^Received a moderate Delphi score after three Delphi rounds but was not voted in during the final Delphi face-to-face meeting |
